# Anti-Adipogenic Effect of *Alchemilla monticola* is Mediated *Via* PI3K/AKT Signaling Inhibition in Human Adipocytes

**DOI:** 10.3389/fphar.2021.707507

**Published:** 2021-08-18

**Authors:** Saveta G. Mladenova, Liliya V. Vasileva, Martina S. Savova, Andrey S. Marchev, Daniel Tews, Martin Wabitsch, Claudio Ferrante, Giustino Orlando, Milen I. Georgiev

**Affiliations:** ^1^ BB-NCIPD Ltd., National Center of Infectious and Parasitic Diseases, Ministry of Health, Sofia, Bulgaria; ^2^ Laboratory of Metabolomics, Department of Biotechnology, Institute of Microbiology, Bulgarian Academy of Sciences, Plovdiv, Bulgaria; ^3^ Department of Plant Cell Biotechnology, Center of Plant Systems Biology and Biotechnology, Plovdiv, Bulgaria; ^4^ Division of Pediatric Endocrinology and Diabetes, Department of Pediatrics and Adolescent Medicine, Ulm University Medical Center, Ulm, Germany; ^5^ Department of Pharmacy, G. D’Annunzio University, Chieti, Italy

**Keywords:** obesity, adipocytes, PI3K, *Alchemilla*, astragalin, quercitrin

## Abstract

Obesity is a persistent and continuously expanding social health concern. Excessive fat mass accumulation is associated with increased risk of chronic diseases including diabetes, atherosclerosis, non-alcoholic steatohepatitis, reproductive dysfunctions and certain types of cancer. *Alchemilla monticola* Opiz. is a perennial plant of the Rosaceae family traditionally used to treat inflammatory conditions and as a component of weight loss herbal mixtures. In the search for bioactive leads with potential anti-adipogenic effect from *A. monticola* extract (ALM), we have employed nuclear magnetic resonance (NMR) based metabolomics to obtain data for the phytochemical profile of the extract. Further, molecular docking simulation was performed against key adipogenic targets for selected pure compounds, present in the ALM extract. Evaluation of the biological activity was done in human adipocytes exposed to ALM (5, 10 and 25 μg/ml), pure astragalin (AST) or quercitrin (QUE) both at the concentrations of 5, 10 and 25 μM. Investigation of the molecular pathways involved was performed through real-time quantitative PCR and Western blot analyses. According to the docking predictions strong putative affinity was revealed for both AST and QUE towards peroxisome proliferator-activated receptor gamma (PPARγ) and phosphoinositide 3-kinase (PI3K). Assessment of the intracellular lipid accumulation revealed anti-adipogenic activity of ALM. Correspondingly, the expression of the adipogenic genes CCAAT/enhancer-binding protein alpha (*CEBPA*) and *PPARG* was downregulated upon ALM and AST treatment. The Western blotting results exposed protein kinase B (AKT), PI3K and PPARγ as targets for the inhibitory effect of ALM and AST on adipogenesis. Collectively, we provide a broader insight of the phytochemical composition of *A. monticola*. Additionally, we demonstrate the anti-adipogenic effect of ALM and its active compound AST in human adipocytes. Furthermore, PI3K/AKT signaling pathway is identified to mediate the ALM anti-adipogenic action. Hence, the ALM extract and its secondary metabolite AST are worth further exploration as potentially active agents in obesity management.

## Introduction

Obesity and obesity-related metabolic syndromes represent a pandemic-scale health threat. Increased adiposity is usually associated with a higher risk for development of chronic diseases such as type 2 diabetes, hypertension, atherosclerosis, dyslipidaemia, non-alcoholic steatohepatitis, reproductive dysfunctions, immune disturbances, chronic low-grade inflammation and certain types of cancer (Vasileva et al., 2018; Arner et al., 2019; Bluher, 2019; Ma et al., 2020). Etiological factors for obesity progression include energy disbalance, genetic susceptibility, neuroendocrine disturbances, environmental and behavioural factors, drug therapy and epigenetic changes (Carayol et al., 2017; Bluher, 2019; Fathzadeh et al., 2020). The adipose tissue is the main fat depot and consists of diverse cell types, including mature adipocytes, preadipocytes, stem cells, endothelial cells, and resident immune cells (Arner et al., 2019; Ramirez et al., 2020). Obesity environment subjects adipose tissue to transformations resulting in increased size (hypertrophy) and number (hyperplasia) of adipocytes, an infiltration by immune cells, and the development of low-grade chronic inflammation and fibrosis (Vasileva et al., 2018). The described changes define different types of stress such as oxidative, metabolic and mechanical existing in the adipose tissue, which trigger the disbalance in intra- and intercellular communication during obesity (Vasileva et al., 2020a; Wang et al., 2021; Zaghlool et al., 2021). Modulation of the adipogenic differentiation is among the most attractive strategies in obesity management (Aprile et al., 2018; Mandl et al., 2019; Rebollo-Hernanz et al., 2019; Vasileva et al., 2021). The process of adipogenic differentiation is tightly regulated by the interaction between multiple transcription factors. Insulin binding to the insulin receptor (INSR) activates insulin receptor substrate 1 (IRS-1) in adipocytes, hence, regulating the initiation of differentiation through two major signaling pathways: the phosphoinositide 3-kinase (PI3K)/protein kinase B (AKT) pathway and the Ras/mitogen-activated protein kinase (MAPK) pathway (Min et al., 2013; Ortega-Molina et al., 2015; Huang et al., 2018; Liu et al., 2019). While the Ras/MAPK signaling pathway is closely associated with proliferation, the PI3K/AKT pathway mediates cellular growth signals and metabolic functions such as glucose utilization and lipid biosynthesis in adipocytes (Lee et al., 2017; Li et al., 2020; Huang et al., 2021). Tyrosine phosphorylation of IRS-1 activates PI3K through interaction with its regulatory subunit p85 ultimately leading to AKT phosphorylation. Subsequently, the activation of AKT stimulates glucose transporter 4 (GLUT4) translocation, increase the glucose influx and the lipogenesis in adipocytes (Lee et al., 2017; Huang et al., 2018; Cignarelli et al., 2019; Liu et al., 2019; Huang et al., 2021). These stimulatory signals are processed by the key players CCAAT-enhancer-binding proteins (C/EBPs), peroxisome proliferator-activated receptors (PPARs), and sterol-regulatory-element-binding protein (SERBP) and their downstream targets responsible for lipid biosynthesis such as fatty acid synthase (FASN) and acetyl-coA carboxylase (ACC) to transform the precursor cell into a mature adipocyte (Kim et al., 2018; Vasileva et al., 2018; Cignarelli et al., 2019; Mandl et al., 2019; Wang et al., 2021).

Medicinal plants have centuries of exploitation, including for weight control, mainly based on an empirical ethnopharmacological knowledge (Vasileva et al., 2018). The multicomponent composition and the chemical complexity of plant extracts contribute to the synergic effect in obesity treatment (Vasileva et al., 2020b; Atanasov et al., 2021). Among the most important advantage of the natural products is that their effect is exerted through modulation in more than one molecular mechanism. Several possible mechanisms of action are explored for the natural anti-obesity compounds such as suppression of appetite, inhibition of lipid absorption, alteration in adipocyte function and differentiation, effect on β-cells and insulin sensitivity, beneficial effect on the gut microbiota and resolution of the obesity-related chronic inflammation (Min et al., 2013; Hussain et al., 2020; Li et al., 2020; Vasileva et al., 2020b; 2021). However, elucidation of the precise mechanism of action of the plant extracts and the active compound or group of compounds involved in their biological effect is critical for the translation of traditional knowledge towards modern therapeutic applications. Deciphering the mechanism of action of medicinal plants with the contemporary “omics” approaches such as metabolomics, transcriptomics and proteomics could accelerate to the discovery of new bioactive leads with anti-obesity potential (Park et al., 2016; Georgiev et al., 2020; Zaghlool et al., 2021).

*Alchemilla monticola* Opiz. (ALM) is a perennial plant traditionally used to treat inflammatory conditions, wounds and burns (Choi et al., 2018; Hwang et al., 2018; Tasić-Kostov et al., 2019). Additionally, a weight loss effect is described for its administration within traditional herbal mixtures (Abbet et al., 2014; Jarić et al., 2015; Masullo et al., 2015; Gras et al., 2017). From a chemical perspective, *Alchemilla* species are known as sources of flavonoids, flavonoids glycosides, phenolic acids and tannins (Choi et al., 2018; Mandrone et al., 2018). The biological properties of *Alchemilla* extracts are reported as cholinesterase inhibitory activity (Neagu et al., 2015), hepatoprotective activity (Ozbek et al., 2017), anti-inflammatory effect against gastric ulcers (Valcheva-Kuzmanova et al., 2019) and photoprotective effect on skin (Hwang et al., 2018). Nevertheless, the potential anti-adipogenic effect of ALM or its pure bioactive compounds in the context of obesity has not been properly addressed.

Here, we have hypothesized the anti-adipogenic potential of ALM extract. Nuclear magnetic resonance (NMR) spectroscopy was applied to identify pure compounds of interest in the ALM extract. An *in silico* molecular docking approach was employed to predict interactions between the selected natural compounds and key adipogenic targets. The biological activity assays of ALM extract and pure astragalin (AST) and quercitrin (QUE) were performed in human adipocytes as an *in vitro* obesity model. Gene and protein expression analyses were completed to shed light on the underlying molecular pathways involved in the anti-adipogenic activity of the ALM extract and its active constituents. In addition, a mechanism of action of the plant extract in human adipocytes is proposed.

## Materials and Methods

### Materials

Apo-transferrin, biotin, Bradford solution, cortisol, dexamethasone, dimethyl sulfoxide (DMSO), 3-(4,5-dimethylthiazol-2-yl)-2,5-diphenyl tetrazolium bromide (MTT), Dulbecco’s modified Eagle’s medium/nutrient F-12 Ham, fetal bovine serum, 4% formalin solution, insulin, 3-isobutyl-1-methylxantine, isopropanol, Oil red O solution (ORO), pantothenic acid, penicillin/streptomycin 10 000 IU/10 mg/ml, protease and phosphatase inhibitor cocktail, RIPA lysis buffer, rosiglitazone, triiodothyronine and 3-(trimethylsilyl)propionic-2,2,3,3-d4 acid sodium salt (TSPA-d4; purity 99%) were supplied from Merck KGaA (Darmstadt, Germany). Pure AST (purity ≥ 95%) and QUE (purity ≥ 95%) were purchased from PhytoLab GmbH and Co. KG (Vestenbergsgreuth, Germany). Deuterated methanol (CD_3_OD; 99.8%) and water (D_2_O; 99.9%) were purchased by Deutero GmbH (Kasbellaun, Germany). All chemicals and reagents for electrophoresis, immunoblotting and real-time quantitative reverse transcription-polymerase chain reaction (RT-qPCR) were purchased from Bio-Rad Laboratories Inc. (Hercules, CA, United States).

### Plant Material and Extraction

Aerial parts from *A. monticola* were collected in 2018 from Rhodope mountain, Bulgaria and voucher specimen (№ 176697) was deposited at the herbarium of the Bulgarian Academy of Sciences. Following freeze-drying on VirTis BenchTop Pro with Omnitronics™ from Genevac Ltd. (Ipswich, United Kingdom) the grounded plant material was extracted in 50% aqueous methanol (1:30 w/v) with ultrasound for 20 min at room temperature. The extract was filtrated, evaporated to dryness under vacuum at 40°C, and stored at −20°C, prior to use.

### NMR-Based Metabolite Profiling

The NMR spectroscopy was performed according to the protocol described elsewhere (Georgiev et al., 2015). The 1H NMR and 2D NMR spectra were recorded at 25°C on a Bruker AVII+ 600 spectrometer (Karlsruhe, Germany), operating at frequency of 600.13 MHz with relaxation time 4.07 s and methanol-d4 as an internal lock.

The resulting 1D and 2D spectra were further manually phased, baseline corrected, and referenced to the internal standard TSPA-d4 at 0.0 ppm by using MestReNova software 12.0.1 from Mestrelab Research (Santiago de Compostela, Spain). The main compounds were assigned to the spectral signals, according to previously published data (Wolfender et al., 2013; Ganbaatar et al., 2016; Mandrone et al., 2018; Marchev et al., 2020).

### Molecular Docking Analysis

Docking calculations were conducted through the Autodock Vina of PyRx 0.8 software. The crystal structures of the target proteins were derived from Protein Data Bank (PDB, www.wwpdb.org) with PDB IDs as follows: 1NWQ for C/EBPα; 2P4Y for PPARγ, 1O6L for AKT, 5ITD for PI3K. In order to prepare the proteins for the docking simulation, all the water molecules and the co-crystalized heteromolecules were removed, followed by addition of hydrogen atoms and neutralization using Kollman united-atom charges. The dimensions of the grid box were 60 × 60 × 60 with 0.375 Å distance between points. Autodock4 and Lamarckian genetic algorithms were used to dock 250 conformations for each test compound (Molinspiration Database, www.molinspiration.com). Discovery studio 2020 Visualizer was employed to investigate the protein-ligand non-bonding interactions.

### Cell Culture and Treatment

The human Simpson-Golabi-Behmel syndrome (SGBS) cells were maintained and differentiated according to the optimal conditions described earlier (Wabitsch et al., 2001; Vasileva et al., 2020b). Following initiation of adipogenic differentiation, the cells were repeatedly exposed to treatment with every culture media renewal on day 0, four and eight respectively. All treatment concentrations were selected upon cell viability evaluation with a MTT assay (Supplementary Figure 1). The ALM extract was added to the culture media at final concentrations of 5, 10 and 25 μg/ml while pure AST and QUE were tested at concentrations of 5, 10 and 25 µM. Vehicle group treated with 0.2% DMSO was included as control. All assays were performed 24 h after the last treatment (on the ninth day of differentiation).

### Intracellular Lipid Accumulation Assay and Free Glycerol Release

Formalin fixed differentiated SGBS adipocytes were stained with ORO solution as described previously (Vasileva et al., 2020b). Total accumulated lipids were quantified by measuring the absorbance of the ORO dye extracted from the adipocytes after staining at 495 nm on Anthos Zenyth 340 multiplate reader from Biochrom Ltd. (Cambridge, United Kingdom).

The free glycerol content released in the culture media was measured with Adipolysis assay kit (#MAK313) from Merck KGaA, following the manufacturer’s instructions.

### RT-qPCR Analysis

Total RNA was isolated with Quick-RNA Miniprep kit from Zymo Research (Irvine, CA, United States) and reverse transcribed using Canvax FirstStrand cDNA kit (Cordoba, Spain). Gene expression of major adipogenic genes was evaluated with RT-qPCR using Sso EvaGreen SuperMix on CFX96 system (Bio-Rad) and was quantified by the comparative threshold cycle method (ΔΔCT) on the CFX Maestro software (Bio-Rad). Ribosomal protein L13a (*RPL13A*) and beta-tubulin (*TUBB*) were employed as reference genes. Primer sequences used for the RT-qPCR are listed in Supplementary Table S1.

### Western Blot Analysis

Total protein lysates were prepared and analyzed through Western blotting as previously described (Vasileva et al., 2021). Equivalent amounts of 30 μg of protein samples were resolved on a sodium dodecyl sulfate polyacrylamide gel and transferred to nitrocellulose blotting membranes. The protein data was normalized over a housekeeping protein on Image Lab 6.0.1 software (Bio-Rad). Antibodies were purchased as follows: rabbit anti-AKT (#9272), anti-C/EBPα (#2295), anti-PI3K (#4257) and anti-PPARγ (#2443) antibodies from Cell Signaling Technology (Leiden, Netherlands); rabbit anti-adiponectin antibody (#GTX112777) from GeneTex Inc. (Ivrine, CA, United States); hFAB rhodamine anti-tubulin (#12004166) and anti-GAPDH (#12004168) antibodies and goat anti-rabbit IgG StarBright Blue 700 (#12004162) secondary antibody from Bio-Rad.

### Statistical Evaluation

The obtained data were analysed with SigmaPlot software v11.0 from Systat Software GmbH (Erkrath, Germany) and presented as mean ± standard error of the mean (SEM). Differences among groups were determined with unpaired Student’s *t*-test where appropriate or one-way analysis of variances (ANOVA) followed by a Bonferroni’s *post hoc* analysis when more than two groups were compared. Values of *p <0.05 were considered for a statistically significant difference.

## Results

### Metabolite Profiling of *A. monticola* Extract

The phytochemical characterization of ALM extract was performed by ^1^H NMR and heteronuclear single quantum coherence spectroscopy (HSQC) profiling. According to the ^1^H NMR spectral data the most abundant signals corresponded to AST (kaempferol-3-O-glucoside), QUE (quercetin-3-O-rhamnoside), catechin, quercetin, isoquercetin, quercetin-3-O-β-glucuronide, rutin, vanillic acid and vitexin (Table 1).

**TABLE 1:** Chemical shifts (δ) and coupling constants (*J*) of *Alchemilla monticola* Opiz. extract metabolites, identified by relevant ^1^H NMR and two-dimensional NMR spectra (Wolfender et al., 2013; Ganbaatar et al., 2016; Mandrone et al., 2018; Marchev et al., 2020).

Metabolite	Chemical shift (δ, ppm)	Multiplicity/coupling constant (*J*, Hz)
Amino acids		
Alanine	1.48	(d, *J* = 7.4)
Threonine	1.33	(d, *J* = 6.5)
Valine	1.00/1.02	(d, *J* = 7.2)/(d, *J* = 7.0)
Sugars		
α-Glucose	5.18	(d, *J* = 3.7)
β-Glucose	4.58	(d, *J* = 7.8)
Sucrose	5.40/4.17	(d, *J* = 3.6)/(d, *J* = 8.7)
Fructose	3.62/3.69/4.03	(m)/(m)/(m)
Organic acids		
Fumaric acid	8.45	(s)
Succinic acid	2.52	(s)
Phenolic acids		
Vanillic acid	7.58/7.55/6.87/3.93	(d, *J* = 2.0)/(dd, *J* = 8.5; 2.2)/(d, *J* = 8.2)/(s)
Flavonoids and flavonoid glucosides		
Astragalin	6.28/6.48/6.97/8.04/5.23	(d, *J* = 2.0)/(d, *J* = 2.0)/(d, *J* = 8.5)/(d, *J* = 8.6)/(d, *J* = 7.8)
Catechin	4.69/4.14/2.82/2.49/6.02/6.03/6.88/6.87/6.79	(d, *J* = 7.4)/(td, *J* = 8.1; 4.3)/(dd, *J* = 16.5; 8.3)/(dd, *J* = 16.0; 8.7)/(d, *J* = 2.0)/(d, *J* = 2.0)/(d, *J* = 2.0)/(d, *J* = 8.2)/(dd, *J* = 8.4; 2.3)
Isoquercitrin	6.28/6.48/7.68/6.97/7.55/5.23	(d, *J* = 2.0)/(d, *J* = 2.0)/(d, *J* = 2.0)/(d, *J* = 8.5)/(dd, *J* = 8.5; 2.2)/(d, *J* = 7.8)
Kaempferol	6.28/6.48/7.00/8.04	(d, *J* = 2.0)/(d, *J* = 2.0)/(d, *J* = 8.5)/(d, *J* = 8.6)
Quercetin	6.28/6.48/7.72/6.97/7.64	(d, *J* = 2.0)/(d, *J* = 2.0)/(d, *J* = 2.0)/(d, *J* = 8.5)/(dd, *J* = 8.5; 2.3)
Quercetin-3-O-β-glucuronide	7.73/7.55/6.87/6.42/6.28/5.23	(d, *J* = 2.0)/(dd, *J* = 8.5; 2.2)/(d, *J* = 8.2)/(d, *J* = 2.2)/(d, *J* = 2.2)/(d, *J* = 7.8)
Quercitrin	6.28/6.42/7.31/7.00/7.55/5.270.97	(d, *J* = 1.8)/(s)/(d, *J* = 2.0)/(d, *J* = 8.5)/(dd, *J* = 8.4; 2.0)/(d, *J* = 1.6)/(d, *J* = 6.3)
Rutin	6.28/6.48/7.68/6.97/7.64/5.06/4.54	(d, *J* = 2.0)/(d, *J* = 2.0)/(d, *J* = 2.0)/(d, *J* = 8.5)/(dd, *J* = 8.8; 2.4)/(d, *J* = 8.2-Glu)/(d, *J* = 2.0-Rha)
Vitexin	6.69/6.37/8.00/7.92/7.00/5.10	(s)/(s)/(d, *J* = 8.9)/(s)/(d, *J* = 8.5)/(d, *J* = 10.1)
Others		
Inositol	3.91/3.61/3.20	(m)/(m)/(m)
γ-Aminobutyrate	1.90/2.32/3/01	(m)/(t, *J* = 7.5)/(t, *J* = 7.5)

The structures of some of the molecules such as AST and QUE have been elucidated also by the signals observed in both ^1^H NMR and HSQC spectra (Supplementary Figure 2). In the proton NMR and HSQC spectrum of ALM extract, several signals typical of aromatic hydrogens were observed: two doublets at *δ*_H_ 6.28 and *δ*_H_ 6.46, which were correlated with the carbon resonance at *δ*_C_ 101.66 and *δ*_C_ 97.02 corresponded to a *meta*-coupling of H6 and H8 protons, attributed to the flavonoid A-ring. Another two doublets with *ortho*-coupling constants at *δ*_H_ 6.97 and *δ*_H_ 8.04 were correlated with *δ*_C_ 118.36 and *δ*_C_ 131.33 and corresponded to H3’/5’ and H2’/6’, suggesting two *para*-substituted B-rings of flavonoids. The anomeric proton (H1’) of glucose appeared at *δ*_H_ 5.23/*δ*_C_ 105.12. Compared to previously published data (Rezende et al., 2019) it could be considered that the structure corresponds to AST.

In QUE, the *δ*_H_ 5.27/*δ*_C_ 93.36 suggested the presence of α-linked rhamnose moiety. The meta-coupled protons of A-ring (H6 and H8) of the flavonoid nucleus were recognized according to the HSQC correlations *δ*_H_ 6.28/*δ*_C_ 101.66 and *δ*_H_ 6.42/*δ*_C_ 97.02. Signals at *δ*_H_ 7.31/ *δ*_C_ 110.98, *δ*_H_ 7.00/ *δ*_C_ 117.98 and *δ*_H_ 7.55/ *δ*_C_ 125.02 were assigned to H2’, H5’ and H6’ of the ring B, which suggested a QUE nucleus (Wolfender et al., 2013).

We have selected AST and QUE for further *in silico* and *in vitro* evaluation of their anti-adipogenic potential based on the results of the ALM metabolite profiling. In addition, the previously reported data on AST and QUE biological activity related to lipid metabolism directed our interest towards these two compounds identified in the ALM extract (Roman Junior et al., 2015; Hur et al., 2020; Hussain et al., 2020; Swamy et al., 2020).

### *In Silico* Docking Simulation

**FIGURE 1:**
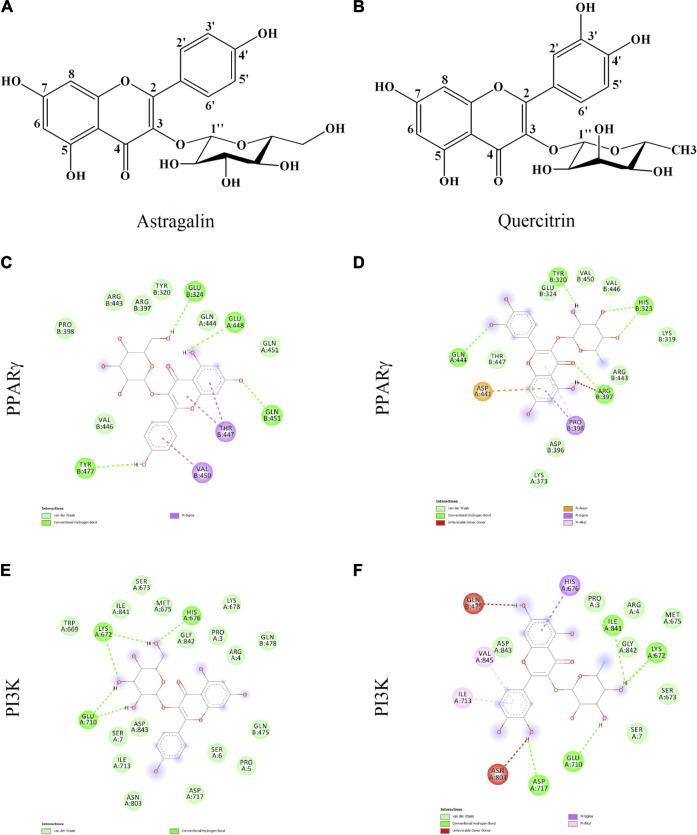
Astragalin and quercitrin were predicted to exert strong affinity towards peroxisome proliferator-activated receptor gamma (PPARγ) and phosphoinositide 3-kinase (PI3K). Astragalin chemical name: 5,7-dihydroxy-2-(4-hydroxyphenyl)-3-[(2S,3R,4S,5S,6R)-3,4,5-trihydroxy-6-(hydroxymethyl)oxan-2-yl]oxychromen-4-one; Molecular weight 448.38 g/M **(A)**. Quercitrin chemical name: 2-(3,4-dihydroxyphenyl)-5,7-dihydroxy-3-[(2S,3R,4R,5R,6S)-3,4,5-trihydroxy-6-methyloxan-2-yl]oxychromen-4-one; Molecular weight 464.38 g/M **(B)**. Potential interactions between: astragalin and PPARγ **(C)**; quercitrin and PPARγ **(D)**; astragalin and PI3K **(E)**; quercitrin and PI3K **(F)**.

Docking runs were performed in order to predict the putative affinities of AST and QUE towards the following target proteins: C/EBPα, PPARγ, AKT and PI3K. The affinity of the selected phenolic compounds with the aforementioned target proteins were expressed in terms of binding free energy (_∆_G) and binding constant (Ki; Table 2). All predicted affinities were in the micromolar range (0.02–24.4 µM). However, the lowest binding constant values of both AST and QUE were towards PI3K and PPARγ. Therefore, the orientation of AST and QUE at the protein binding sites with these two protein targets are presented in Figure 1. The strong binding energies (from −7.3 to −9.2 kcal/M) suggested a potential implication of both PI3K and PPARγ in the mechanism of action of AST and QUE in adipocytes.

**TABLE 2:** Free energy of binding (_Δ_G, kcal/M) and affinity (Ki, µM) for each component to protein structure.

Target protein	Astragalin	Quercitrin
AKT (PDB: 1O6L)	−6.7 kcal/M; 12.4 µM	−6.8 kcal/M; 10.5 µM
C/EBPα (PDB: 1NWQ)	−6.3 kcal/M; 24.4 µM	−6.3 kcal/M; 24.4 µM
PI3K (PDB: 5ITD)	−9.2 kcal/M; 0.2 µM	−8.7 kcal/M; 0.4 µM
PPARγ receptor (PDB: 2P4Y)	−7.3 kcal/M; 4.5 µM	−7.8 kcal/M; 1.9 µM

### *A. monticola* Extract Reduced Lipid Accumulation in Human Adipocytes

**FIGURE 2:**
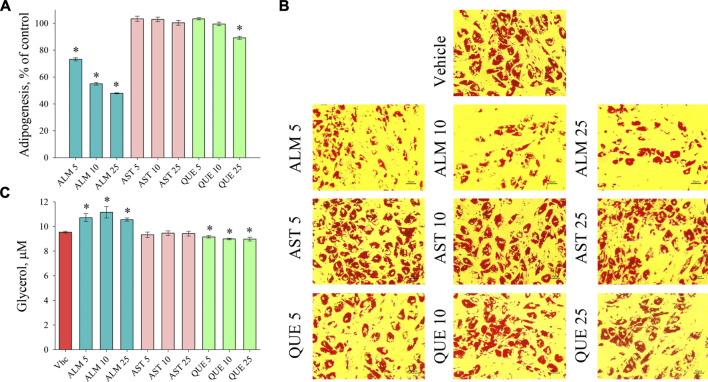
*Alchemilla monticola* Opiz. (ALM) reduced lipid accumulation and promoted basal lipolysis in human adipocytes. Levels of adipogenesis presented as percentage of vehicle control group of adipocytes treated with ALM 5, 10 and 25 μg/ml, astragalin (AST) 5, 10 and 25 μM or quercitrin (QUE) 5, 10 and 25 μM from the Oil red O assay **(A)**. Representative images (magnification ×20; scale bar 50 μm) of all experimental groups following the Oil red O staining **(B)**. Evaluation of basal lipolysis through free glycerol release in the culture media **(C)**. Data are expressed as mean ± SEM; each experimental group consisted of at least six technical replicates from three independent biological experiments. *p < 0.05 compared to vehicle control group.

*De novo* adipogenic differentiation of preadipocytes to mature fat cell is a hormonal-dependent process initiated predominantly by insulin signaling characterized with increasing intracellular lipid accumulation (Arner et al., 2019; Cignarelli et al., 2019; Mandl et al., 2019). Assessment of the accumulated lipids with ORO assay revealed a significant anti-adipogenic activity of the ALM extract (Figures 2A,B). Moreover, application of the ALM extract was found to increase basal lipolysis, which was reflected by the increased free glycerol released in the culture media (Figure 2C). Interestingly, neither the adipogenesis nor the lipolysis processes were affected upon the AST treatment (Figure 2). Regarding the QUE application, a significant decrease in accumulated lipids was observed only at the highest concentration used (Figures 2A,B) and the levels of lipolysis were moderately decreased (Figure 2C). These findings suggest that the ALM extract possess anti-adipogenic potential, which could be at least partly dependent on the presence of AST and QUE in the extract.

### *A. monticola* Extract, AST and QUE Downregulated Key Adipogenic Genes

**FIGURE 3:**
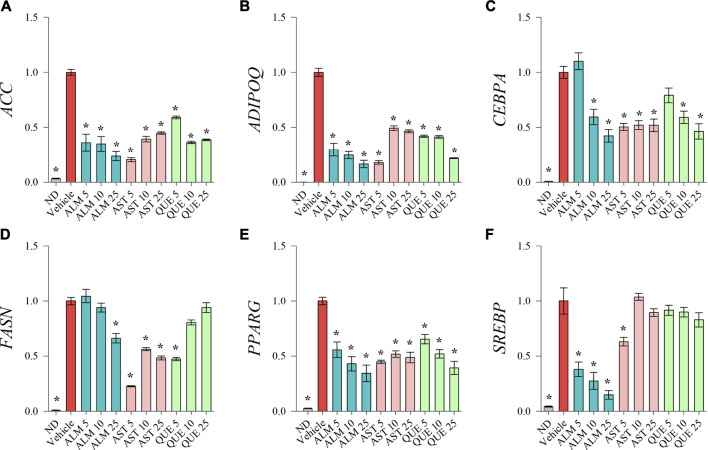
Treatments with *Alchemilla monticola* Opiz. (ALM) extract, astragalin (AST) and quercitrin (QUE) suppressed adipogenic gene expression in human SGBS adipocytes. Relative mRNA expression normalized to vehicle control group (_∆∆_Cq) for acetyl-coA-carboxylase **(** *ACC*; **A)**, adiponectin **(** *ADIPOQ*; **B)**, CCAAT/enhancer-binding protein alpha **(** *CEBPA*; **C)**, fatty acid synthase **(** *FASN*; **D)**, peroxisome proliferator-activated receptor gamma **(** *PPARG*; **E)** and sterol regulatory element-binding protein **(** *SREBP*; **F)** from the RT-qPCR analysis. Three replicates were performed from three independent biological repeats. Data are presented as mean ± SEM. *p < 0.05 versus the vehicle control group.

To investigate the underlying molecular pathways involved in the anti-adipogenic effect of ALM and its pure compounds we have evaluated the mRNA expression levels of key adipogenic factors and genes from the lipid biosynthesis by RT-qPCR (Figure 3). Expression of *CEBPA* (Figure 3C) and *PPARG* (Figure 3E) was downregulated upon ALM, AST and QUE treatment in human SGBS adipocytes. Further, *ADIPOQ* (Figure 3B) and *SREBP* (Figure 3F) were dose-dependently suppressed by the application of ALM extract. The genes *ACC* (Figure 3A) and *FASN* (Figure 3D) were significantly downregulated as a result of ALM suggesting disruption of the lipogenic pathways. Interestingly, expression levels of *ACC, ADIPOQ, FASN* genes were negatively affected upon AST and QUE administration, but *SREBP*, their upstream regulator was not influenced.

Taken together, the gene expression analysis points out the dose-dependent inhibitory effect of ALM extract on adipogenic gene transcriptional activation. Despite the fact that AST and QUE did not affected lipid accumulation on the ORO assay (Figure 2), the results of the RT-qPCR suggest that both compounds interfere with the genetic regulation during adipogenesis. Therefore, we could speculate that AST and QUE are involved in the observed anti-adipogenic effect of the ALM extract.

### *A. monticola* Disrupted PI3K/AKT Signaling Pathway and Negatively Regulated PPARγ and C/EBPα

Adipogenic differentiation is initiated in the preadipocyte upon stimulation by external signals –such as insulin, cortisol or other growth factors. The INSR transmits the insulin signal through IRS phosphorylation and activate the PI3K/AKT pathway to induce pro-adipogenic transcription factors which direct the cell fate towards adipogenesis (Ortega-Molina et al., 2015; Huang et al., 2018; Liu et al., 2019; Huang et al., 2021). Thereafter, mainly C/EBPα and PPARγ govern the firm regulation of the process of adipogenic differentiation (Liu et al., 2014; Park et al., 2016; Kim et al., 2018; Vasileva et al., 2021). Their overexpression leads to subsequent activation of SREBP, which in turn elevate lipid biogenesis through ACC and FASN, ultimately leading to increase in the formation of lipid droplets. Simultaneously, adiponectin levels are increasing as a major adipogenic marker of the mature adipocyte (Liu et al., 2014; Kim et al., 2018; Arner et al., 2019; Xu et al., 2020).

**FIGURE 4:**
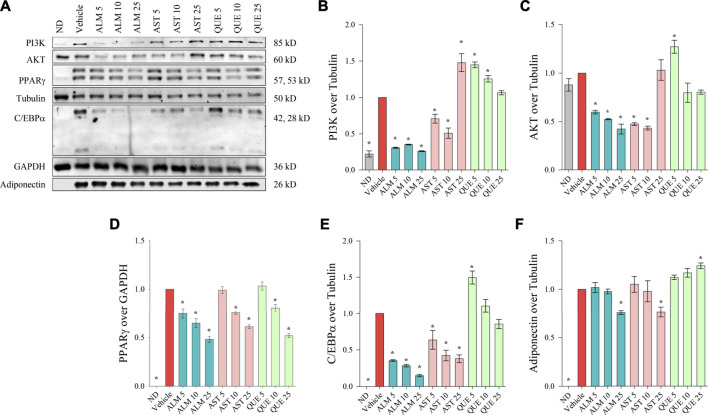
*Alchemilla monticola* Opiz. (ALM), astragalin (AST) and quercitrin (QUE) diminished the protein levels of key adipogenic factors in human adipocytes. Representative images from the Western blot analysis **(A)**. Relative protein levels of phosphoinositide 3-kinase **(**PI3K; **B)**, protein kinase B **(**AKT; **C)**, peroxisome proliferator-activated receptor gamma (PPARγ; **D**), CCAAT/enhancer-binding protein alpha **(**C/EBPα; **E)**, and adiponectin **(F)**. Tubulin or GAPDH were used as housekeeping proteins. Results are expressed as mean ± SEM and are representative of three independent experiments. *p <0.05 compared to the vehicle control group.

To further elucidate the molecular mechanism of action of ALM and its pure active compounds AST and QUE we have evaluated the protein levels of PPARγ, C/EBPα and adiponectin as key adipogenic markers by Western blotting. Considering the crucial role of PI3K/AKT signaling in adipocyte differentiation and the predicted affinity of AST and QUE towards PI3K and AKT from the *in silico* analysis, we have detected their protein abundance as well (Figure 4A).

We have observed that ALM extract negatively affect both PI3K (Figure 4B) and AKT (Figure 4C) in a dose-dependent manner, hence, supporting the observed results from the docking simulations, the ORO assay and the RT-qPCR. Intriguingly, the effect of AST on these two targets appeared bidirectional. The lower concentrations of AST (up to 10 μM) induced decrease in PI3K and AKT levels whereas the highest concentration elevated both proteins. In contrast, PI3K and AKT levels were mildly elevated as a result of the exposure to QUE.

Consistently to the mRNA expression analysis, a dose-dependent decline in the protein abundance of PPARγ, C/EBPα and adiponectin was determined upon ALM treatment (Figure 4). In addition, AST was found to inhibit PPARγ (Figure 4D) and C/EBPα (Figure 4E) in a manner corresponding to the observed effect on their respective genes (Figures 3C,E). However, regarding adiponectin (Figure 4F) AST moderately decreased its levels at the highest concentration of 25 μM. Interestingly, QUE positively affected the adiponectin protein abundance (Figure 4F) and mildly inhibited PPARγ only at the highest concentration (Figure 4D).

Altogether, these results indicated that ALM constrained the process of adipogenesis through interfering the PI3K/AKT signaling pathway and thus inhibiting the adipogenic markers PPARγ, C/EBPα and adiponectin. Further, both AST and QUE affected PPARγ, C/EBPα and adiponectin abundance, which supports the notion that these proteins play a role in the anti-adipogenic action of the extract.

### Proposed Mechanism of the Anti-Adipogenic Action of *A. monticola* Extract

**FIGURE 5:**
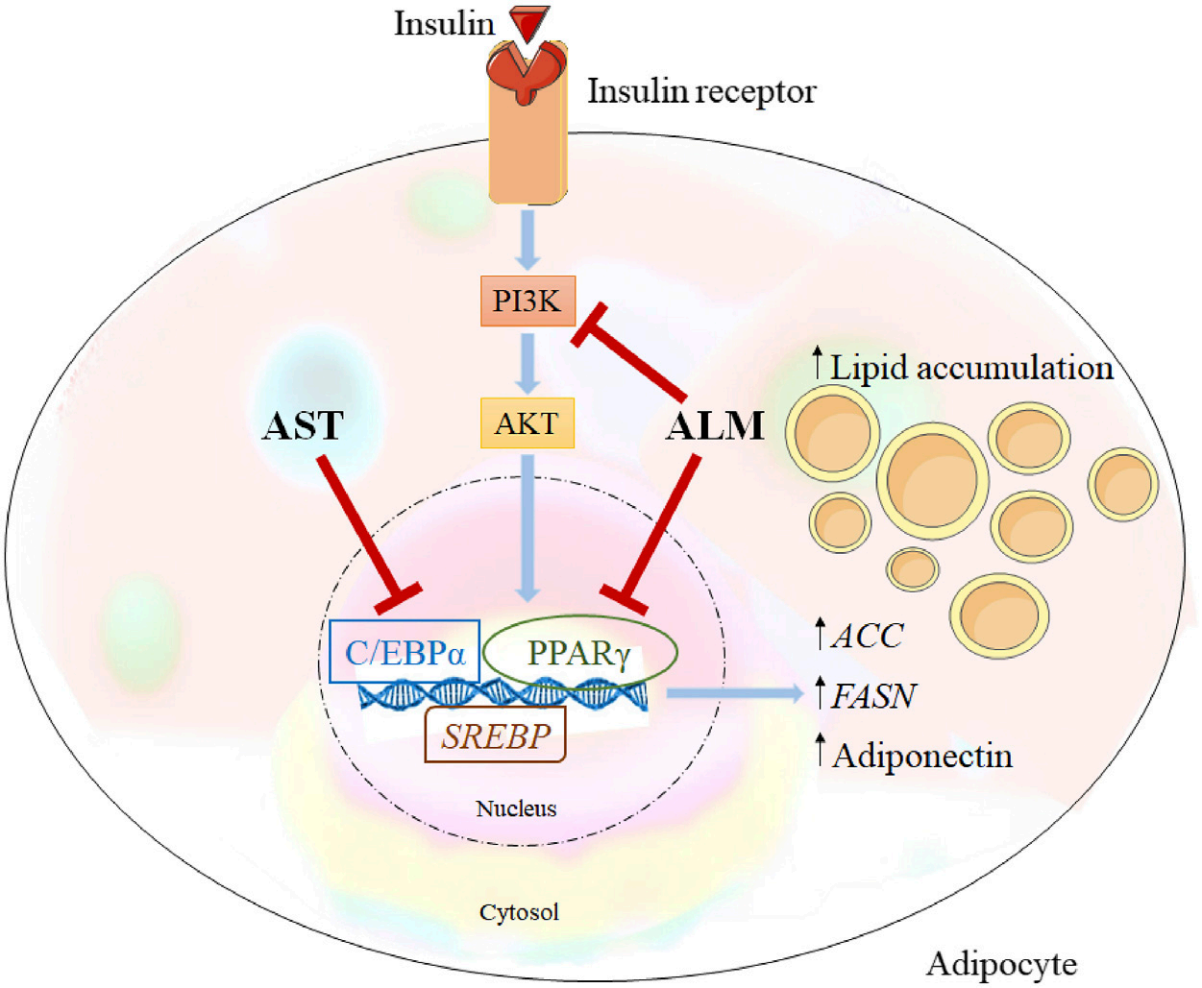
*Alchemilla monticola* Opiz. (ALM) and astragalin (AST) proposed mechanism of action. Inhibitory effect of ALM on adipogenesis is simultaneously through inhibition of phosphoinositide 3-kinase/protein kinase B (PI3K/AKT) signalling and peroxisome proliferator-activated receptor gamma (PPARγ), CCAAT/enhancer-binding protein alpha (C/EBPα) as important adipogenic regulators. In contrast, AST suppressed predominantly C/EBPα.

Integration of the obtained data from the docking predictions, the gene expression analysis and alternations in protein levels provided evidence of the potential mechanisms of the anti-adipogenic effect of ALM and its pure compound AST (Figure 5). Western blotting results revealed that ALM extract anti-adipogenic effect is most probably mediated through inhibition of the PI3K/AKT signaling pathway. Additionally, direct inhibitory activity of the ALM towards C/EBPα and PPARγ protein could be suggested which subsequently leads to decrease in adiponectin. Similarly, pure AST negatively affects C/EBPα and PPARγ and to a lesser extend the adiponectin, PI3K and AKT. Therefore, it could be speculated that AST anti-adipogenic effect is associated rather with direct interaction with PPARγ or C/EBPα than with modulation of PI3K.

## Discussion

Obesity development correlates with the regulation of body metabolism by fat tissue function. Therefore, it is of great importance to identify bioactive leads that target adipocyte function to prevent and cure this complex metabolic disease. The PI3K/AKT signaling pathway in adipocytes is responsible for initiation of differentiation and lipid biosynthesis (Liu et al., 2019; Li et al., 2020; Huang et al., 2021). One of the primary substrates for AKT-related lipid metabolism is SREBP, which regulates fatty acid biosynthesis and cholesterol-related genes (Huang et al., 2018; Rebollo-Hernanz et al., 2019). Once preadipocytes are involved in the process of adipogenic transformation, the expression of PPARγ and C/EBPα activates a transcriptional cascade that induces the expression of genes associated as adipocyte markers, such as ACC, FASN, adiponectin, etc. (Mandl et al., 2019; Vasileva et al., 2021).

A number of studies have demonstrated the biological properties of *Alchemilla* extract, including hepatoprotective (Ozbek et al., 2017), antioxidant (Neagu et al., 2015), anti-inflammatory (Choi et al., 2018; Valcheva-Kuzmanova et al., 2019) and photoprotective activity (Hwang et al., 2018). For example, administration of 200 mg/kg body weight of *Alchemilla* extract protected the liver tissue from carbon tetrachloride-induced toxicity (Ozbek et al., 2017). Currently, the potential anti-adipogenic mechanism of ALM administration has not been clearly addressed.

Flavonoids and their derivatives are a class of natural compounds with abundance within the plant kingdom that exert versatile bioactivity including anti-diabetic and anti-obesity effects (Lee et al., 2017; Hussain et al., 2020; Li et al., 2020; Huang et al., 2021). The beneficial properties of the flavonoids and flavonoid glycosides in diabetes and obesity management are achieved *via* modulation in targeted signaling networks such as the IRS/PI3K/AKT/GLUT4 pathway, thereby improving glucose metabolism, and glucose transport or aldose reductase by carbohydrate metabolic pathways in pancreatic β-cells, hepatocytes, adipocytes and skeletal myofibers (Goto et al., 2012; Lee et al., 2017; Hur et al., 2020; Hussain et al., 2020; Li et al., 2020; Wu et al., 2020; Huang et al., 2021). However, a frequently reported obstacle for the use of flavonoids *in vivo* is their limited bioavailability (Hussain et al., 2020). Several recent studies reported greater bioavailability and enhanced bioactivity of the flavonoid glycosides than their respective aglycone (Lee et al., 2017; Huang et al., 2021). For example, Lee et al. (2017) compared the anti-adipogenic effect of quercetin and quercetin-3-O-glucoside both *in vitro* and *in vivo*. The authors provided evidence that the glycoside form inhibits the adipogenic differentiation and lipogenesis more effectively than the aglycone through potent PPARγ inhibition in adipocytes and enhanced lipid β-oxidation in liver tissue (Lee et al., 2017). The NMR-based metabolite profiling identified ALM extarct as a source of various flavonoids and flavonoid glycosides, mainly quercetin and kaempferol glycosides, such as AST and QUE among others. Many of the identified compounds in the ALM extract, including quercetin, isoqercitrin, quercetin-3-O-glucoside, kaempferol and catechin have been sufficiently investigated for their effect in different *in vitro* and *in vivo* obesity and diabetes models (Lee et al., 2017; Hussain et al., 2020; Huang et al., 2021) Therefore, we have selected AST and QUE as these two compounds appeared in a small number of studies that suggest their anti-adipogenic potential. Moreover, studies on the effect of AST and QUE on the adipogenic differentiation in human adipocytes were insufficient. Several previous studies reported that AST regulated lipogenesis and fat accumulation in 3T3-1L adipocytes *via* suppression of the mRNA expression of PPARγ, C/EBPα, FASN and leptin (Swamy et al., 2020; Wu et al., 2020). Similarly, QUE demonstrated significant effect against liver fat accumulation and decreased inflammatory markers in different liver injury models (Roman Junior et al., 2015; Hur et al., 2020). Based on their presence in the ALM extract and the data suggesting potential to influence lipid accumulation in several experimental models, we have targeted for our present investigation AST and QUE.

The present investigation revealed that ALM extract could inhibit the differentiation and lipid accumulation of human adipocytes, and its active components AST and QUE are at least partly involved in the observed anti-adipogenic effect. The ALM extract reduced the expressions of various adipogenesis-related genes and proteins and inhibited the differentiation of human preadipocytes. Mechanistically, the ALM extract suppressed PI3K/AKT-mediated signaling cascade, which triggers proliferation of preadipocytes and initiate adipogenic program, and this inhibitory effect could be attributable to the inhibition of INSR kinase or IRS by direct binding of AST or other ALM compound to active PI3K. The protein levels of PI3K and AKT were dose-dependently reduced upon ALM treatment. However, consistently with the docking predictions the mechanistic assays showed that pure AST had bidirectional effects on the PI3K/AKT signaling (inhibition at low concentrations up to 10 μM and activation at high concentration). This indicates that AST may have a dose-related bidirectional regulation effect on the PI3K/AKT signaling pathway of human adipose cells, and the critical dose of this bidirectional regulation is between 10 and 25 μM. Similarly, the anti-adipogenic effect of cocoa polyphenols in murine adipocytes and mice with diet-induced obesity has been confirmed in previous study (Min et al., 2013). The authors have provided evidence that cocoa polyphenols directly inhibit the INSR and subsequently suppressed both ERK- and PI3K/AKT-mediated signaling cascades (Min et al., 2013).

Insulin signaling in the adipose tissue has a critical role in lipid storage as well as glucose homeostasis. In the presence of insulin, the INSR autophosphorylates and subsequently phosphorylates proteins of the IRS family, thereby activating two main signaling pathways, including Ras/MAPK and PI3K/AKT pathways (Huang et al., 2018; Liu et al., 2019; Rebollo-Hernanz et al., 2019; Li et al., 2020; Huang et al., 2021). Activation of IRS-1 promotes phosphorylation of AKT, activated by a binding between the regulatory subunit of PI3K and phosphotyrosine residues on IRS-1 (Cignarelli et al., 2019; Liu et al., 2019; Rebollo-Hernanz et al., 2019). Consequently, the PI3K/AKT signaling pathway participates in the initiation of differentiation through complex signaling cascades (Ortega-Molina et al., 2015; Huang et al., 2018; Li et al., 2020; Huang et al., 2021). Therefore, the inhibitory effect of ALM on PI3K kinase activity and its downstream AKT signaling pathways appears to mediate its anti-adipogenic effect. Given to the key role of IRS-1 in the PI3K/AKT pathway activation, a potential interaction of ALM or its some of its compounds with IRS-1 could also be involved in the observed PI3K/AKT inhibition. However, the strong binding affinity of both AST and QUE towards PI3K revealed by our molecular docking data and the significant suppression in protein abundance of PI3K upon ALM application rather points out PI3K as the most probable direct target of the ALM extract in adipocytes.

Тhe PI3K/AKT pathway is also responsible for a wide range of metabolic functions in other tissues (Huang et al., 2018; Cignarelli et al., 2019; Rebollo-Hernanz et al., 2019; Xu et al., 2020). Therefore, blocking INSR activity should be employed under careful consideration as it could produce detrimental outcomes such as insulin resistance development. Previous studies have demonstrated that INSR activity is defective in the skeletal muscle of obese and diabetic humans, and liver specific INSR knockout mice exhibit severe insulin resistance and glucose intolerance (Min et al., 2013; Li et al., 2020; Xu et al., 2020). In this regard, pure AST that acts as an PI3K inhibitor can possibly trigger insulin resistance. Finally, although activation of INSR by insulin is a major inducer of adipogenesis, the insulin-like growth factor (IGF)-1 and the IGF-1 receptor (IGF1R) are other critical factors to initiate insulin signaling by activating PI3K/AKT-mediated signaling cascades (Huang et al., 2018; Cignarelli et al., 2019). Thus, it may be possible that AST, QUE or other ALM components interact with IGF1R, leading to the inhibition of adipogenesis without diminishing insulin action. Further studies are required to investigate whether ALM extract or its pure compounds protect against the development of insulin resistance by increasing IGF1R activity or whether it exerts direct inhibitory effect on INSR is involved. However, we could not determine whether ALM or AST improves insulin resistance in the present study as we use an *in vitro* model system. Moreover, another possible explanation could be that the suppression of PI3K/AKT signaling observed upon ALM and pure AST treatment is tissue specific, hence, potential effect on insulin sensitivity may differ between different cell types.

The transcription factors PPARγ and C/EBPα are continuously expressed in mature adipocytes (Aprile et al., 2018; Kim et al., 2018; Vasileva et al., 2021). In addition to the modulation of PI3K/AKT signaling, the ALM extract and its pure components hamper the differentiation of adipocytes reflected by reduction in the expression of PPARγ and C/EBPα at both gene and protein levels. Interaction of both AST and QUE with PPARγ and/or C/EBPα were revealed by the *in silico* simulation and further confirmed by the gene and protein expression analysis. In a recent study, Swamy et al. (2020) have demonstrated that AST (kaempferol-3-O-glucoside) negatively regulates adipogenesis through inhibition in PPARγ, C/EBPα, FASN and leptin. Consistent with this, our findings revealed a concentration-dependent decrease upon AST application, which had a stronger effect on C/EBPα protein abundance in comparison to PPARγ. Hence, C/EBPα appears as a potential direct target for the AST action in adipocytes. Interestingly, despite the structural similarity between AST and QUE and the close values of the affinity constants predicted from the docking study, the suppression of both PPARγ and C/EBPα protein abundance from QUE was mild to moderate. The protein abundance of PPARγ was inhibited only by QUE at its highest concentration of 25 μM. In a similar *in vitro* model, quercetin at concentrations up to 20 μM did not induced changes in PPARγ or C/EBPα protein level while its 3-O-glucoside inhibited concentration-dependently both proteins at concentrations of 10 and 20 μM (Lee et al., 2017). Another study evaluating the effect of quercetin on adipogenic differentiation reported inhibition on lipid accumulation at concentrations of 20 and 40 μM and no changes in *PPARG, CEBPA* mRNA expression by quercetin 40 μM (Li et al., 2020). These data and our findings suggest that both the aglycone and the sugar moiety are important for the biological activity of the quercetin glycosides. In addition, concentrations above 20 μM of QUE (quercetin-3-O-rhamnoside) should be used in order to induce significant changes in the adipogenic transcription factors. However, as a component of the ALM, it could be speculated that QUE plays a role in the anti-adipogenic action of the extract by participating in possible synergistic interactions with other components.

Finally, this study provides a theoretical basis for the anti-adipogenic effect of ALM extract mediated through modulation in PI3K/AKT signaling pathway and PPARγ protein levels. The identified pure compounds AST and QUE demonstrated potential for anti-adipogenic action through modulation of different signaling pathways. To further determine these pharmacological effects of ALM extract and its pure components, studies on other cell types and *in vivo* obesity models need to be performed.

In summary, the present investigation demonstrates that ALM inhibits early stage of adipogenesis in human adipocytes through suppression in PI3K activity *via* direct binding. The ALM extract also exhibits anti-adipogenic activity which is attributable to decrease in intracellular lipids and enhanced lipolysis. Altered PPARγ, C/EBPα and adiponectin observed upon ALM treatment in human adipocytes may be associated with the PI3K/AKT signaling inhibition. Additionally, both AST and QUE interfered with the studied adipogenic pathways, however, to a different degree. Collectively, these findings suggest a potential of ALM to prevent obesity that worth further *in vivo* validation. Moreover, the identified phytochemical composition of the ALM extract holds great promise for elucidation of novel bioactive leads targeting at obesity management.

## Data Availability

The original contributions presented in the study are included in the article/Supplementary Material, further inquiries can be directed to the corresponding author.
