# Supplementary material for: Anti-Adipogenic Effect of Alchemilla monticola is Mediated Via PI3K/AKT Signaling Inhibition in Human Adipocytes
Source: Front Pharmacol. 2021 Aug 18;12:707507. doi: 10.3389/fphar.2021.707507 (PMC8416315; doi:10.3389/fphar.2021.707507)
Supplement: Supplementary file 1 [file DataSheet1.docx]

Supplementary Material

# Supplementary Figures and Tables

## Supplementary Figures

**
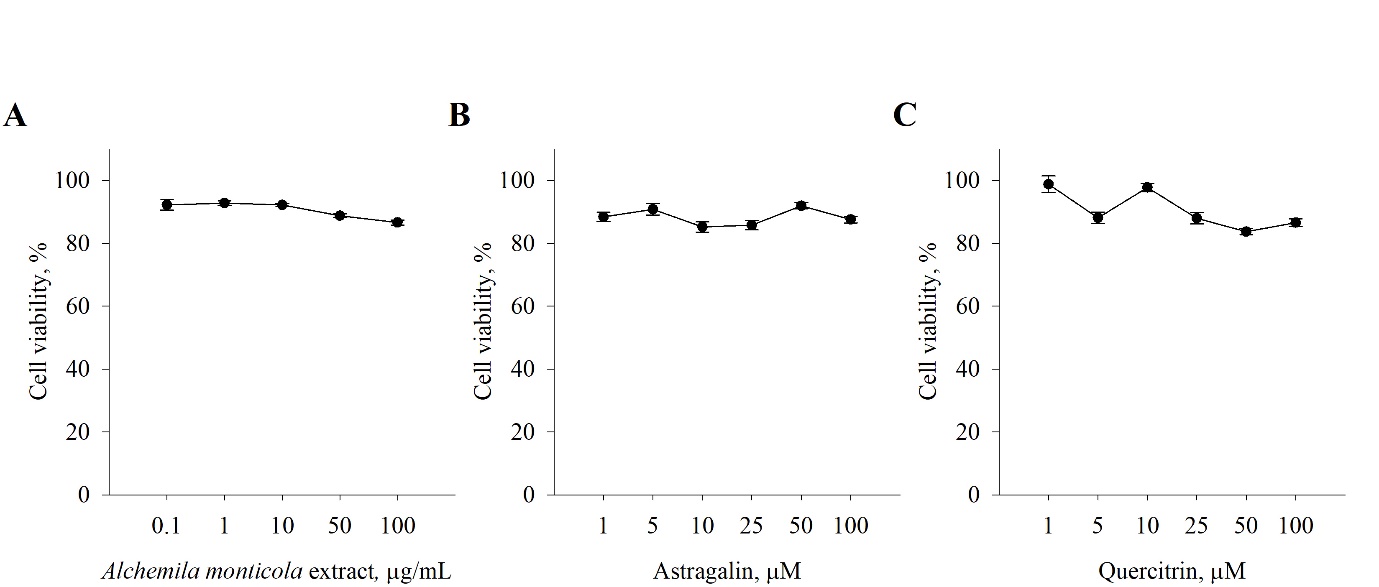
**

**Supplementary Figure 1.** Cell viability of SGBS cells after 24 h of treatment with increasing concentrations of *Alchemilla monticola* Opiz. extract (**A**), astragalin (**B**) and quercitrin (**C**)*.* Results are shown as mean ± SEM based on three replications. Statistically significant difference as compared to vehicle control was presented with p < 0.05. In order to confirm that A. *monticola* aerial parts extract and its active substances astragalin and quercitrin have not effect on the cell viability, MTT assay was performed. The confluent preadipocytes were treated with *A. monticola* extract (concentration range 0.1-100 μg/mL), astragalin and quercitrin (concentration range 1-100 μM). After 24 h incubation 10 μL volume of 5 mg/mL MTT solution was added to each well for 3 h. Absorption was measured at 570 nm wavelength and presented as a percentage of viable cells in treated groups versus the vehicle group. In addition, vehicle treated group showed that 0.2 % DMSO do not affect cell viability in SGBS cells. The *A. monticola* aerial parts extract, astragalin, quercitrin and DMSO up to the highest experimental concentrations do not influence cell viability.


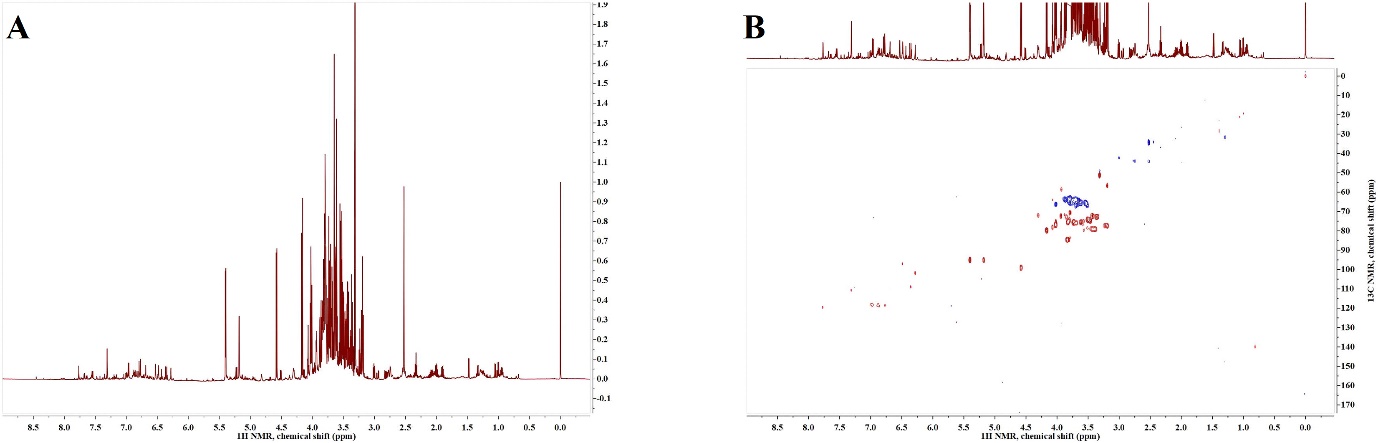


**Supplementary Figure 2.** Representative NMR spectra of *Alchemilla monticolla* Opiz. extract. ^1^H NMR (600 MHz) spectra (**A**) and heteronuclear single quantum coherence spectroscopy (^1^H-^13^C HSQC) spectra (**B**).

## Supplementary Tables

**Supplementary Table 1.** Primer pairs designed for the real-time quantitative PCR.

| **Target gene (human)** | **Forward primer (5’ - 3’)** | **Length** | **Reverse primer (5’ - 3’)** | **Length** | **Tm f/r (°C)** | **Product length (bp)** |
| --- | --- | --- | --- | --- | --- | --- |
| *ACC* | TTCACTCCACCTTGTCAGCG | 20 | GTCAGAGAAGCAGCCCATCA | 20 | 60.25/59.75 | 99 |
| *ADIPOQ* | TGCCCAAAGAGGAGAGAGGAA | 21 | TCAGAAACAGGCACACAACTCA | 22 | 60.49/60.36 | 97 |
| *CEBPA* | TATAGGCTGGGCTTCCCCTT | 20 | CTAGGTCTCCCTCTCCCACC | 20 | 60.03/60.11 | 148 |
| *FASN* | TCTACGGCTCCACGCTCTT | 19 | GAAGAGTCTTCGTCAGCCAGG | 21 | 60.68/60.40 | 130 |
| *PPARG* | GATCCAGTGGTTGCAGATTACAA | 23 | GAGGGAGTTGGAAGGCTCTTC | 21 | 58.99/60.07 | 144 |
| *RPL13A* | AAAAGCGGATGGTGGTTCCT | 20 | GCTGTCACTGCCTGGTACTT | 20 | 59.89/59.96 | 118 |
| *SREBP1* | TGTACTTCTGGAGGCATCGC | 20 | CTACAAGCCAGGTCCAGGTG | 20 | 59.82/60.04 | 139 |
| *TUBB* | AGCCGTCTTACTCAACTGCC | 20 | GTCACCCAGAATGGCAGAA | 19 | 60.04/59.96 | 198 |

Abbreviations: bp, base pairs; f, forward; r, reverse; Tm, melting temperature.
